# Key Factors Driving Physiotherapy Use in Patients with Nonspecific Low Back Pain: Retrospective Clinical Data Analysis

**DOI:** 10.3390/jcm13206261

**Published:** 2024-10-20

**Authors:** Dalia M. Alimam, Muteb J. Alqarni, Mawaddah H. Aljohani, Mohammed A. Alqarni, Abdulrahman M. Alsubiheen, Asma S. Alrushud

**Affiliations:** 1 Department of Health Rehabilitation Sciences, College of Applied Medical Sciences, King Saud University, P.O. Box 10219, Riyadh 11433, Saudi Arabia; 2 Physiotherapy Department, Royal Commission Medical Center, Yanbu 46451, Saudi Arabia

**Keywords:** low back pain, physiotherapy use, clinical factors, retrospective study

## Abstract

**Background/objectives**: Understanding the factors that influence physiotherapy (PT) service use among patients with nonspecific lower back pain (LBP) is necessary to optimize treatment strategies, healthcare resource allocation, and the planning of value-based initiatives. We report factors that influence the number of PT visits per episode of care (defined as a referral from a physician) for an LBP population in Saudi Arabia, and compare them with patients experiencing their first and recurrent episodes of LBP. **Methods**: LBP patients were retrospectively enrolled from a clinical dataset derived from an outpatient PT clinic in Saudi Arabia. The primary outcome variable was the number of PT visits performed per episode of care. Multiple linear regression analysis was performed to examine the relationships between the numbers of PT visits per episode of care and independent variables. **Results**: The number of PT sessions per week (β 0.34, *p* < 0.001), compliance with PT sessions (β 0.31, *p* < 0.001), and pre-pain scores (β 0.29, *p* < 0.001) explained 41.8% (adjusted R^2^ 0.32) of the variance in the total number of PT visits per episode of care (*p* < 0.001). **Conclusions:** Factors that might improve value-based care for LBP patients are reported. The more PT sessions per week, compliance with these sessions, and higher baseline pain scores predict a higher number of PT visits per episode of care among these patients. While reported for a Saudi Arabian population, there is no reason to believe that these findings do not apply internationally.

## 1. Introduction

Nonspecific low back pain (LBP) is a significant global problem that affects individuals of varied socioeconomic status and demographics [1]. The LBP burden extends beyond mere physical distress because it influences an individual’s daily activities, work-related activities, social participation, and overall quality of life [1,2,3]. The economic implications of LBP are also substantial, with direct healthcare costs and indirect costs caused by lost productivity placing a considerable burden on healthcare systems and society as a whole [4].

The multifactorial nature of LBP, in that its underlying causes range from mechanical to psychosocial, complicates its management [5,6,7,8]. While pain itself is seldom attributed to a specific anatomical abnormality, its impact on individuals can be extreme, leading to repeated healthcare use [3,9]. Physiotherapy (PT) is the most prevalent treatment approach, with various interventions, such as exercise therapy, manual therapy, and education, aimed at relieving pain, improving function, and preventing recurrence [10,11].

Value-based healthcare encourages providers, including physiotherapists, to optimize resource use [12,13]. By focusing on evidence-based practices and efficient treatment protocols, physiotherapists can decrease the number of unnecessary visits and treatments that do not contribute significantly to patient outcomes [12,13]. Because neither the number of visits nor the money spent on healthcare predict better outcomes for people with musculoskeletal health conditions [14,15], an improved understanding of the factors that influence PT use among nonspecific LBP patients is needed to optimize treatment strategies, healthcare resource allocation, and the planning of effective value-based initiatives [16,17,18,19].

Enhancing PT treatment strategies requires insight into factors that influence patient decisions, including beliefs about treatment efficacy, accessibility of services, and personal experiences [16,17,18,19]. For instance, factors such as previous PT by the same healthcare provider, self-discharge from PT, and therapist certification (e.g., manual therapist) all predict more PT visits. Higher out-of-pocket payments also significantly reduce the number of visits per episode of care [19]. These factors may vary in different cultural and organizational settings.

In Saudi Arabia, there is a lack of established care pathways and local clinical guidelines for LBP management [20]. Patients can consult general practitioners at primary healthcare centers or pay to see spine specialists in private clinics, but referral wait times for PT can reach up to four months [20,21]. Due to this lack of established care pathways, physiotherapists managing LBP may consult international guidelines, leading to variability in treatment approaches and the number of prescribed sessions [22,23,24]. The lack of evidence-based practice implementation among physiotherapists [22] is another regional challenge that may be associated with increased PT visits per episode of care. Although direct access to PT is now permitted (i.e., private clinics), accessibility remains limited, particularly in public hospitals, which delay the initiation of PT services for LBP at an early stage [20,25,26]. Early initiation of physiotherapy services could facilitate early recovery and lead to subsequent decreases in consultation and healthcare costs. The general population in Saudi Arabia (SA) also has an average level of awareness and knowledge of PT services [27].

The timing of PT for LBP affects health service use and cost [28,29]. Early PT is associated with fewer chiropractic, pain specialist, emergency department, and orthopedist visits, as well as lower opioid use and fewer epidural steroid injections [29]. This suggests that timely intervention is important to manage acute LBP [30], and that doing so will also reduce longer-term demands on healthcare resources and associated costs. Recurrent LBP also leads to increased use of diagnostic imaging services and higher overall medical expenses [31]. Therefore, investigating these factors in different settings can inform healthcare providers on how to develop value-based services more strategically, improve patient outcomes, and optimize healthcare resource allocation to manage nonspecific LBP through PT [32]. Accordingly, our objectives are to identify those factors that influence the number of physiotherapy visits per episode of care for the nonspecific LBP population in Saudi Arabia, and, secondarily, to identify and compare key factors in individuals experiencing LBP for the first time with those in individuals experiencing recurrent LBP.

## 2. Methods

### 2.1. Study Design

A retrospective cross-sectional study was performed on a clinical dataset derived from an outpatient PT clinic of the Royal Commission for Yanbu in Saudi Arabia.

### 2.2. Population and Sample

Dataset participants were screened from 300 LBP case files from the clinic. For study inclusion, participants had to be ≥18 years of age and have been transferred to and received outpatient PT for nonspecific LBP between 1 August 2021 and 31 March 2023. All diagnoses were confirmed by a physician prior to patient referral to the physiotherapy department, ensuring that appropriate management plans were implemented. Typical physiotherapy care included a combination of education, manual therapy, heat therapy, therapeutic exercises, and electrotherapy. Patients who had reported (1) features of serious pathology (e.g., malignancy, infection, cauda equina syndrome), (2) specific pathologies (e.g., radiculopathy), (3) a history of back surgery, or (4) pregnancy were excluded from the study.

### 2.3. Data Source

Anonymized patient data were extracted from the provider’s electronic health records. Physiotherapist staff enter data into this system. In the event that more than one therapist had provided care to a patient, the therapist of record was the therapist who performed the initial examination. One raw data file containing anonymized variables on a secure electronic Excel sheet was provided.

### 2.4. Study Variables

#### 2.4.1. Outcome

The primary outcome variable was the number of PT visits performed per episode of care, defined as a referral from a physician for an LBP issue. Each of the patient’s visits to the physiotherapist (a patient encounter) was documented. Each episode of care began on the date the physiotherapist performed the initial examination and ended on the date on which a discharge note was completed. When a discharge note was unavailable, the last visit date—defined as a visit that was not followed by another physiotherapist visit for the same patient’s file within 30 days—was used; 30 days was chosen based on work demonstrating that 95% of patients with acute LBP returned to work within 4 weeks [33].

To avoid partial episodes of care [19], any that began before 1 August 2021 or after 31 January 2023, without the generation of a discharge note, were excluded.

#### 2.4.2. Explanatory Variables

Explanatory variables were chosen following Dolot et al. [19], and according to the data available in the database. Variables were categorized following Anderson and Newman’s behavioral model of health service use [34]. This model recognizes the importance of individual and healthcare system factors governing a person’s health services use. Variables were subclassified into individual factors (e.g., age, sex), individual’s need (e.g., date of first PT session, time gap between doctor’s referral and first PT session, number of PT sessions), clinical-related factors (e.g., duration of LBP, occurrence of LBP, the presence of other medical problems such as hypertension), and PT-related outcomes (e.g., pre- and posttreatment pain levels, perceived functional improvement, LBP patient’s compliance, and physiotherapist’s years of experience and gender) [35].

Data were presented according to whether the LBP was reported as a first or recurrent episode—an important distinction to recognize factors that influence PT visits during the first LBP episode vs. factors that influence recurrent episodes, and to tailor recommendations or interventions to meet a patient’s specific needs [36]. While we focus on PT use, patient preferences (including factors such as medication use) can also influence decisions regarding the use of alternative treatments [37]. As such, a general question about the medication used for each episode of care was reported as (yes) or (no).

Patient-reported outcomes are regularly used in clinical practice to evaluate treatment outcomes and to guide further interventions [38]. However, in the available dataset, perceived functional improvement was reported as (yes) or (no) and was determined by a patient’s response to the question, (Is there any improvement in your lower back pain)? We also included variables such as the date of the doctor’s referral and the date of the first PT session [39]. Although available data included the clinic’s location, specific policy or resource variables related to the healthcare system were not recorded. Accordingly, we did not use organization variables or the category of healthcare system in the analyses.

### 2.5. Data Analysis

All statistics were performed using the SPSS statistical software package version 25 (IBM Corp., New York, NY, USA). Descriptive statistical analysis was performed using means and 95% confidence intervals for continuous variables, and frequencies and percentages for categorical variables. The total number of PT visits was calculated for patients for each episode of care (first or recurrent), and the factors that influenced the number of PT visits were determined.

Data were assessed using the Shapiro–Wilk test for normal distribution. Continuous data were deemed to be not normally distributed at *p* < 0.05. Where appropriate, Mann–Whitney U, Crosstabs, and Chi-squared tests were used to evaluate the differences between first-time and recurrent LBP patients for each episode of care.

Correlations between the numbers of physiotherapy visits per episode of care and independent variables were determined using Spearman’s rho correlation tests and point-biserial correlation. The correlation coefficient (r) was interpreted as follows: very strong ≥ 0.7, strong 0.5–0.7, moderate 0.3–0.5, weak 0.1–0.3, and no correlation < 0.1 [40].

Categorical variables were transformed into dummy variables for inclusion in the regression models. After creating dummy variables for categorical predictors, multiple linear regression analysis was performed to examine the relationships between the numbers of PT visits per episode of care and independent variables; β coefficients and adjusted R^2^ scores were reported [41]. Cases with missing outcome data were retained for analysis because excluding them could lead to bias (the remaining dataset may not be representative of the entire population) and significantly reduce the dataset size, potentially impacting the study’s statistical power and generalizability [42,43].

## 3. Results

Eligibility reduced the number of files to 183, of which 163 represented first-episode-of-care patients. The mean patient age was 43 y (standard deviation (SD) = 14.2), 143 (78.1%) were female, and 68 (37.2%) patients were categorized as obese. A total of 158 (86.3%) patients were not in paid employment. There were significant between-group differences in age and gender, with most patients experiencing their first episode of LBP. Patient characteristics and individual-related factors are presented in Table 1.

Complete clinical information regarding the sample is presented in Table 2. Of the total sample, 108 (60.6%) cases were categorized as chronic LBP. Most (138; 75.4%) patients reported having taken LBP-related medications.

PT-related factors are presented in Table 3. The mean number of PT treatment sessions per episode of care was 5.2 (SD 2.57). The total number of PT sessions did not differ significantly between patients experiencing their first or recurrent episodes of LBP (*p* = 0.892). The mean time gap between referral and the first PT session was 9.14 (SD 10.02) days, and it was higher among patients with recurrent episodes of care (mean 11.15 d; SD 10.62). The number of PT sessions per week did not differ significantly between the groups (*p* = 0.78). Patients with recurrent LBP reported more previous PT sessions than patients having their first episode (*p* ≤ 0.001). For all patients, the mean pretreatment (baseline) visual analog scale (VAS) pain score was 5.82 (SD 1.35), and the mean posttreatment VAS pain score was 2.03 (SD 1.61). The treating physiotherapists were mainly female and had an average of 4.85 (SD 2.39) years of experience (Table 4).

A statistically significant moderate correlation was apparent between the outcome variable (number of PT visits performed per episode of care) and the number of PT sessions per week, and there was a weak correlation with baseline pain scores. Negative moderate correlations were identified with posttreatment VAS scores and with completed PT sessions. A significant negative correlation was found between the number of PT visits performed per episode of care and perceived functional improvement (Table 5).

The multiple regression model identified several statistically significant variables that explained the total number of PT visits per episode of care. Of these, the number of PT sessions per week (β 0.34, *p* < 0.001), compliance with PT (β 0.31, *p* < 0.001), and baseline pain scores (β 0.29, *p* < 0.001) were the most important, collectively explaining 41.8% (adjusted R^2^ 0.32) of the variance in the total number of PT visits per episode of care (*p* < 0.001) (Table 6). These results indicate that a 0.34-point increase in the number of PT sessions per week, and an increase of 0.31 points in the patient’s compliance with PT would result in a 1-point increase in the total number of PT visits per episode of care. The baseline pain score was associated with a higher number of PT visits per episode of care that was 0.29 units higher than the overall mean.

Separate regression analyses were performed for patients who reported their first episode of LBP and those with recurrent LBP. The multiple regression models for those who had experienced their first episode of LBP revealed factors including number of PT sessions per week (β 0.30, *p* < 0.001), higher baseline pain scores (β 0.28, *p* < 0.001), compliance with PT (β −0.33, *p* < 0.001), and perceived functional improvement (β −0.14, *p* < 0.05) to collectively explain 36.7% (adjusted R^2^ 0.34) of the variance in the total number of PT visits per episode of care (*p* < 0.001). These results indicate that with a 0.30-point increase in the number of PT sessions per week and an increase of 0.28 points in the patient’s baseline pain score, there would be a 1-point increase in the total number of PT visits per episode of care. Higher compliance with PT sessions and an increased perception of functional improvement were associated with fewer PT visits per episode of care. For those patients with recurrent LBP, only the number of PT sessions per week was identified as a factor, accounting for 45.5% (adjusted R^2^ 0.26) of the variance in the total number of PT visits per episode of care (*p* < 0.001) (Appendix A).

## 4. Discussion

This study identifies the factors that should be considered when targeting value-based care for patients with nonspecific LBP. A combination of factors explained 41.8% of the variance in the PT visits per episode of care.

In previous studies, higher out-of-pocket payments per visit [19,44], active PT treatment [45], and earlier use of PT relative to the onset of symptoms [46] have all been reported to predict fewer visits per episode of care for LBP patients [44]. Longer average PT visit length, having previously received PT from the same organization, and a higher baseline physical function have also all been related to a reduced number of PT visits per episode of care [19]. Additionally, the organizational factor (therapist certification) (e.g., certified manual therapist) was reported to increase the number of PT visits per episode of care [19,47]. Differences in identified factors may be related to differences in studied populations, geography, and/or culture, to data quality and sources (e.g., self-reported vs. retrospective), or simply to the inclusion of different variables in studies.

In cultures where out-of-pocket expenses may be regarded as major barriers to accessing care, people might be less likely to seek frequent treatment [48,49]. In this study, a higher number of PT sessions per week, compliance with PT visits, and higher baseline pain scores predicted a greater number of PT visits per episode of care among LBP patients. This may be explained by the therapeutic protocol used, because patients with higher baseline pain scores may believe that they require more intensive PT therapy [50,51,52], and seek more PT sessions. Patients who attend PT more frequently may be more motivated and compliant with their treatment plan [53]. Higher compliance rates can also lead to more comprehensive treatment plans [54], with therapists prescribing multiple sessions per week to achieve specific therapeutic goals more quickly [28,54,55,56], thereby increasing the total number of PT visits per episode of care. Unfortunately, such a treatment protocol may hinder the development of self-management skills, which are essential for the long-term management of nonspecific LBP [57,58]. Thus, a vicious cycle of overreliance on frequent PT sessions can develop, resulting in unnecessary treatment that limits access to resources for others and encourages low-value practices [59,60].

It is important to support value-based care to promote a patient’s engagement and self-management, thereby empowering them and leading to health improvement that is more sustainable. There is an increasing need to adhere to clinical practice guidelines, to improve the cost-effectiveness of care and clinical outcomes, and to reduce costs and visits by directing clinicians toward management strategies that are more effective [6]. One recommendation was to use patients’ reported outcome measurements (PROMs) in clinical practice to support patient care [61,62]. Although the great majority (95.5%) of Saudi physiotherapists reported using patient-reported outcomes [63], most outcomes involved a unidirectional assessment of pain intensity (i.e., VAS). Consistent with our findings, more multidirectional and functionally related outcome measures are needed.

For those experiencing their first episode of LBP, we report an additional factor (perceived functional improvement) to be associated with fewer PT visits per episode of care. This is not unexpected, because during the first episode of LBP a physiotherapist may usually focus on educating a patient about self-care strategies (e.g., exercises). Once patients have successfully learned and applied these techniques, they may require fewer PT sessions. Additionally, some demographic characteristics may be related to functional recovery (e.g., younger age, being a nonsmoker, and having a shorter duration of LBP) [64,65]. Accordingly, the self-management of LBP should be encouraged to reduce the number of PT visits to align with the principle of value-based care.

Clinically, these results may encourage healthcare providers to adjust the frequency of PT sessions according to patient needs, concomitantly enhancing physiotherapy service efficiency. To achieve this, it is important to develop strategies that improve patient compliance with PT sessions. Such strategies may involve sharing the therapeutic decision (where the patient and therapist agree on a treatment plan that is based upon the patient’s preferences and the therapist’s knowledge of evidence-based practice), the setting of clear and realistic goals, evidence-based education, and the use of motivational techniques to encourage compliance with PT sessions. These results may also encourage clinicians to incorporate additional multidimensional PROMs to assess functional recovery and to enhance patient-centered care. For researchers, clinical guidelines may need to integrate recommendations regarding session frequency to optimize resource use.

This study has several limitations, primarily its retrospective nature, which may introduce biases in data collection and analysis. Additionally, the original dataset did not contain PROMs to assess functional improvements, which could have provided valuable insights into patient perspectives and experiences.

Our findings provide novel insights into the challenges and dynamics of PT use in Saudi Arabia, emphasizing the need for established care pathways and evidence-based practices among physiotherapists. Specifically, we explore the potential of timely physiotherapy interventions for LBP to reduce healthcare utilization, an area not previously examined to the best of our knowledge. By highlighting the importance of self-management in LBP, our study advocates for changes in healthcare practices to improve patient outcomes in Saudi Arabia.

## 5. Conclusions

While previous studies have identified predictors for a higher number of PT sessions per episode of care, we have identified new factors that are deemed to be of importance, at least for the population of Saudi Arabia. These include the frequency of PT sessions per week, patient compliance with these sessions, and baseline pain scores. Excessive dependence on frequent sessions can impede the development of self-management skills. Therefore, it is essential to strike a balance between session frequency and the promotion of self-management strategies to enhance overall treatment effectiveness. While we report these for a Saudi Arabian population, there is no reason to believe that they would not apply more universally. The implications of these findings certainly extend to domestic (and potentially international) healthcare managers and policymakers involved with healthcare service delivery and efficiency and healthcare practice patterns.

## Figures and Tables

**Table 1:** Characteristics of study participants (individual-related factors) (n = 183).

Variable		Mean (SD) (95% CI)	No. (%)	1st Episode (n = 163) Mean (SD) (95% CI)	Recurrent (n = 20) Mean (SD) (95% CI)	*p*-Value
Age		43.02 (14.22) (40.95–45.10)		41.94 (14.04) (39.77–44.12)	51.80 (12.92) (45.75–57.85)	0.004 *
Gender	Male		40 (21.9)	32 (80)	8 (2)	0.038 *
	Female		143 (78.1)	131 (91.60)	12 (8.39)	
BMI (n = 175)		27.66 (26.44–28.88)				0.227
	Underweight		11 (6)	10 (90.90)	1 (9.09)	0.272
	Normal weight		44 (24)	42 (95.45)	2 (4.54)	
	Overweight		52 (28.4)	43 (82.69)	9 (17.30)	
	Obese		68 (37.2)	60 (88.23)	8 (11.76)	
Marital status	Single		43 (23.5)	42 (97.67)	1 (2.32)	0.090
	Married		136 (74.3)	118 (86.76)	18 (13.23)	
	Other		4 (2.2)	3 (75)	1 (25)	
Working status	Professionals **		16 (8.7)	15 (93.75)	1 (6.25)	0.898
	Clerical, sales, and service work and labor and related work		8 (4.3)	7 (87.5)	1 (12.5)	
	Not in paid employment/housewives, trainees, students		158 (86.3)	140 (88.60)	18 (11.39)	

SD, Standard deviation; CI, interval. * Significant at *p* < 0.05. ** Professionals include doctors, lawyers, therapists, etc.; Clerical, sales, and service workers include those operating a keyboard, providing information, producing and recording basic financial and statistical information, engaged in the transportation, storage and purchase of goods, working in retail establishments, supervising retail staff, organizing travel and accommodation, assisting teachers, and providing childcare. Labor and related work involve activities involving effort or exertion.

**Table 2:** Clinically related factors.

Variable	Mean (SD) (95% CI)	No. (%)	1st Episode (n = 163) Mean (SD) (95% CI)	Recurrent (n = 20) Mean (SD) (95% CI)	*p*-Value
Duration of LBP					
Acute		30 (16.85)	27 (17.08)	3 (15)	0.946
Subacute		40 (22.47)	35 (22.15)	5 (25)	
Chronic		108 (60.67)	96 (60.75)	12 (60)	
Medication for LBP					
Yes		138 (75.4)	122 (88.40)	16 (11.59)	0.613
No		45 (24.6)	41 (91.11)	4 (8.88)	
Other medical problems (178)					
None		131 (71.6)	120 (91.60)	11 (35.48)	0.045 *
Yes		47 (25.7)	38 (80.85)	9 (19.14)	

SD: Standard deviation; CI: Confidence interval. Other medical problems include hypertension, high cholesterol, hypothyroidism, diabetes, heart disease, and asthma. * Significant at *p* < 0.05.

**Table 3:** Physiotherapy-related factors.

Variable	Mean (SD) (95% CI)	No. (%)	1st Episode (n = 163), Mean (SD) (95% CI)	Recurrent (n = 20) Mean (SD) (95% CI)	*p*-Value
Time gap between referral and 1st PT session (day)	9.14 (10.02) (7.68–10.60)		9.09 (10.23) (7.43–10.76)	11.15 (10.62) (6.18–16.12)	0.336
Number of PT sessions per week	1.54 (0.54) (1.46–1.62)		1.51 (0.55) (1.42–1.60)	1.50 (0.51) (1.26–1.74)	0.779
Total number of PT sessions/episode	5.19 (2.57) (4.82–5.57)		5.20 (2.75) (4.76–5.65)	4.90 (1.07) (4.40–5.40)	0.892
Had previous PT sessions					
Yes		23 (12.6)	7 (30.43)	16 (69.56)	0.000
No		160 (87.4)	156 (97.5)	4 (2.5)	
Pre-pain (VAS)	5.82 (1.35) (5.62–6.02)		5.84 (1.41) (5.62–6.07)	5.75 (1.16) (5.21–6.29)	0.693
Post-pain (VAS)	2.03 (1.61) (1.80–2.27)		2.06 (1.60) (1.80–2.32)	2.20 (1.44) (1.53–2.87)	0.424
Perceived functional improvement					
Yes		157 (85.8)	140 (89.17)	17 (10.82)	0.914
No		26 (14.2)	23 (88.46)	3 (11.53)	
Compliance with physiotherapy sessions					
Yes		110 (60.1)	99 (90)	11 (10)	0.621
No		73 (39.9)	64 (87.67)	9 (12.32)	

**Table 4:** Characteristics of physiotherapists.

Variable		Mean (SD) (95% CI)	No. (%)
Gender	Male		6 (42.9)
	Female		8 (57.1)
Years of experience		4.85 (2.39) (4.50–5.20)	
Nationality	Saudi		14 (100)

**Table 5:** Correlation between the total number of physiotherapy visits and predictors.

Variable	Characteristics	Physiotherapy Visits/Episode
Age	n	183
	Correlation	0.044
	P	0.558
Gap between referral and 1st session	n	183
	Correlation	−0.021
	P	0.777
Number of PT sessions per week	n	183
	Correlation	0.357
	P	0.000 *
Pre-pain VAS	n	183
	Correlation	0.169
	P	0.022 *
Post-pain VAS	n	183
	Correlation	−0.336
	P	0.000 *
Physiotherapist years of experience	n	183
	Correlation	0.053
	P	0.475
Gender	n	183
	Correlation	0.060
	P	0.419
Occurrence of LBP	n	183
	Correlation	−0.040
	P	0.593
Medications for LBP	n	183
	Correlation	−0.028
	P	0.709
Other medical problem	n	178
	Correlation	0.008
	P	0.919
Had previous PT	n	183
	Correlation	−0.036
	P	0.629
Perceived functional improvement	n	183
	Correlation	−0.232
	P	0.002 *
Complete PT session	n	183
	Correlation	−0.365
	P	0.000 *
	Median (minimum, maximum)	*p* value
BMI categories (n = 175)	5 (2, 23)	0.870
Underweight	6 (2, 6)	
Normal weight	4.5 (2, 13)	
Overweight	5 (2, 23)	
Obese	5 (2, 13)	
Marital status (n = 183)	5 (2, 23)	0.594
Single	5 (2, 23)	
Married	5 (2, 13)	
Other	4 (2, 6)	
Work status (n = 182)	5 (2, 23)	0.276
Professionals	6 (3, 23)	
Clerical, sales, and service work and labor and related work	5 (3, 8)	
Not in paid employment/housewives, trainees, students	5 (2, 13)	
Duration of LBP (n = 178)	5 (2, 23)	0.615
Acute	4 (2, 12)	
Subacute	5 (2, 23)	
Chronic	5 (2, 13)	

* *p* < 0.05.

**Table 6:** Multiple linear regression of outcome on statistically significant predictor variables.

Variable	Unstandardized Coefficients		Standardized Coefficients	t	Sig	95.0% Confidence Interval for B	
	B	SE				LB	UB
(Constant)	1.858	1.809		1.027	0.306	5.431	1.715
PT sessions/week	1.651	0.321	0.348	5.141	0.000	1.017	2.286
Compliance with PT	1.628	0.400	0.311	4.070	0.000	0.838	2.418
Pre VAS	0.557	0.139	0.291	4.014	0.000	0.283	0.831

Dependent Variable: Total number of PT; SE, Standard Error; LB, lower bound; UB, upper bound.

## Data Availability

Data are unavailable due to privacy or ethical restrictions.

## Appendix A

**Table A1:** Multiple linear regression of outcomes on non-significant predictor variables.

Variable	Unstandardized Coefficients		Standardized Coefficients	t	Sig.	95.0% Confidence Interval for B	
	B	Std. Error				Lower Bound	Upper Bound
(Constant)	1.858	1.809		1.027	0.306	5.431	1.715
Age	0.002	0.017	0.011	0.120	0.904	0.036	0.032
Female	0.086	0.521	0.014	0.166	0.868	0.943	1.116
Normal	0.088	0.672	0.015	0.131	0.896	1.239	1.415
Overweight	0.216	0.643	0.038	0.336	0.738	1.054	1.485
Obese	0.341	0.639	0.064	0.533	0.595	0.922	1.604
Married	0.042	0.505	0.007	0.083	0.934	0.955	1.039
Other	1.536	1.228	0.088	1.251	0.213	3.962	0.890
Clerical	0.930	0.957	0.074	0.971	0.333	2.821	0.961
Not in paid employment	0.873	0.592	0.117	1.474	0.142	2.042	0.296
Subacute	0.870	0.531	0.140	1.639	0.103	0.179	1.918
Chronic	0.834	0.473	0.160	1.764	0.080	0.100	1.768
First episode	0.994	0.760	0.121	1.308	0.193	0.508	2.496
Taking medications	0.120	0.399	0.020	0.300	0.765	0.669	0.908
No other medical problems	0.202	0.416	0.036	0.487	0.627	0.619	1.024
The time gap between referral and 1st session	0.016	0.017	0.063	0.948	0.344	0.018	0.050
No previous PT	1.173	0.735	0.152	1.596	0.113	2.626	0.279
Perceived functional improvement	0.513	0.564	0.070	0.910	0.364	0.601	1.626
Post VAS	0.142	0.148	0.089	0.962	0.337	0.435	0.150

VAS: Visual Analog Scale.

**Table A2:** Multiple linear regression analysis for the first episode of low back pain.

Variable	Unstandardized Coefficients		Standardized Coefficients	t	Sig	95.0% Confidence Interval for B	
	B	Std. Error				Lower Bound	Upper Bound
(Constant)	0.680	0.869		0.783	0.435	1.036	2.397
Number of PT sessions per week	1.493	0.319	0.302	4.672	0.000	0.862	2.124
Pre VAS	0.559	0.134	0.283	4.171	0.000	0.294	0.824
Post VAS	0.066	0.153	0.040	0.429	0.669	0.368	0.236
Functional improvement	1.154	0.590	0.149	1.958	0.052	2.319	0.010
Compliance	1.848	0.421	0.335	4.394	0.000	2.679	1.017

VAS: Visual Analog Scale.

**Table A3:** Multiple linear regression analysis for recurrent low back pain.

Variable	Unstandardized Coefficients		Standardized Coefficients	t	Sig.	95.0% Confidence Interval for B	
	B	Std. Error				Lower Bound	Upper Bound
(Constant)	2.886	1.391		2.075	0.057	0.097	5.869
Number of PT sessions per week	1.033	0.430	0.495	2.400	0.031	0.110	1.956
Pre VAS	0.210	0.269	0.229	0.784	0.446	0.365	0.786
Post VAS	0.372	0.210	0.4980-	1.767	0.099	0.823	0.080
Functional improvement	0.142	0.596	0.048	0.238	0.816	1.419	1.136
Compliance	0.208	0.573	0.099	0.363	0.722	1.021	1.438

VAS: Visual Analog Scale.
